# P-2160. Cumulative Incidence of Invasive Fungal Disease Among Patients with Multiple Myeloma who Received B-cell Maturation Antigen–Directed CAR T-cell therapy: A Systematic Review and Meta-Analysis

**DOI:** 10.1093/ofid/ofaf695.2323

**Published:** 2026-01-11

**Authors:** Stephanos Vassilopoulos, Athanasios Vassilopoulos, Abby London, Markos Kalligeros, Eleftherios Mylonakis

**Affiliations:** Warren Alpert Medical School of Brown University, Rhode Island Hospital, Providence, RI, Providence, RI; Warren Alpert Medical School of Brown University, Rhode Island Hospital, Providence, RI, Providence, RI; Department of Medicine, Warren Alpert Medical School of Brown University, Rhode Island Hospital, Providence, Rhode Island; Harvard Medical School, Beth Israel Deaconess Medical Center, Boston, MA, Boston, Massachusetts; Houston Methodist Hospital, Houston, TX, Houston, Texas

## Abstract

**Background:**

Patients with multiple myeloma are at higher risk for infections due to disease pathophysiology and administered therapies. The purpose of this study was to estimate the incidence of invasive fungal infections in patients with multiple myeloma who received B-cell maturation antigen (BCMA)–directed chimeric antigen receptor (CAR) T-cell therapy.

**Figure:**
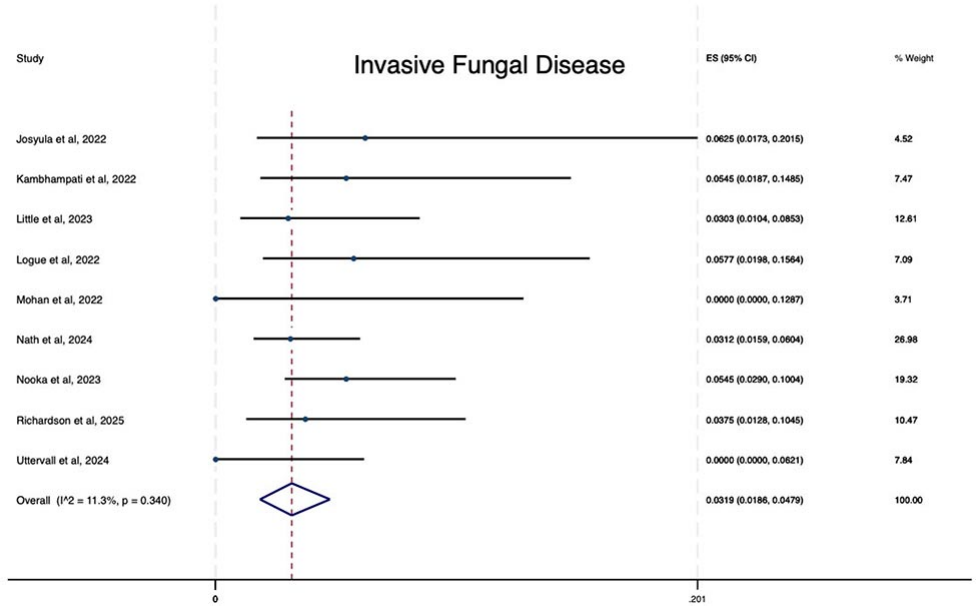
Cumulative incidence rate of IFD among patients with multiple myeloma who received BCMA–directed CAR T-cell therapy

**Figure:**
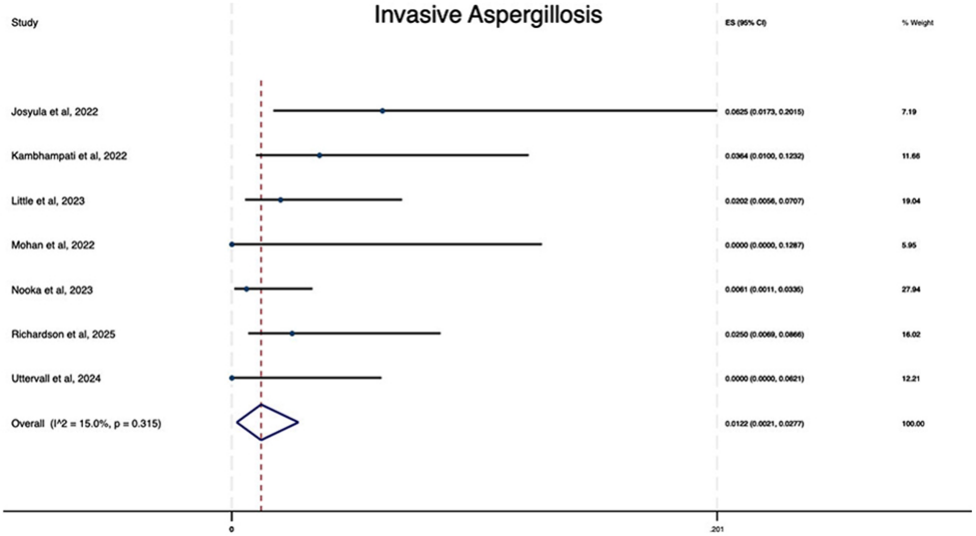
Cumulative incidence rate of invasive aspergillosis among patients with multiple myeloma who received BCMA–directed CAR T-cell therapy

**Methods:**

We searched PubMed and EMBASE for studies that included patients with multiple myeloma who received BCMA–directed CAR T-cell therapy and evaluated the incidence of infections among this patient population. We present the pooled cumulative incidence of invasive fungal disease (IFD), invasive fungal aspergillosis, and invasive fungal candidiasis by performing a random-effects meta-analysis.

**Results:**

After screening 232 citations and excluding studies which did not report infectious complications or with a follow up of less than 30 days, we identified and analyzed studies reporting IFD, invasive aspergillosis, or invasive candidiasis among participants with multiple myeloma who received BCMA-directed CAR T-cell therapy. Specifically, we retrieved 9 studies that reported data on the development of IFD and among 823 patients who received BCMA-directed CAR T-cell therapy, we found that 31 patients developed IFD with a cumulative incidence of 3.19% (95% CI, 1.86-4.79). In addition, we retrieved 7 studies that reported data on the development of invasive aspergillosis, among 515 patients who received BCMA-directed CAR T-cell therapy 9 patients developed invasive aspergillosis with a cumulative incidence of 1.22% (95% CI, 0.21-2.77). For invasive candidiasis, we retrieved 7 studies and among 515 patients who received BCMA-directed CAR T-cell therapy 4 patients developed invasive candidiasis with a cumulative incidence of 0.51 (95% CI, 0.00-1.67).

**Conclusion:**

In this first meta-analysis evaluating the incidence of IFD in patients with MM receiving BCMA-targeted CAR T-cell therapy, we found that this patient population faces a discernible burden of IFD, including aspergillosis and candidiasis. Although these infections remain relatively uncommon, considerable morbidity and mortality among this patient population supports the need for prospective studies aimed at identifying risk factors.

**Disclosures:**

Eleftherios Mylonakis, MD, PhD, Chemic Labs/Koda Therapeutics, LLC: Grant/Research Support|Lumen: DSMB|NIH/NIAID: Grant/Research Support|Sciclone: Grant/Research Support|Shionogi: Advisor/Consultant|Synexis: Clinical trial

